# Arbuscular Mycorrhizal Fungi and the Need for a Meaningful Regulatory Plant Protection Product Testing Strategy

**DOI:** 10.1002/etc.5400

**Published:** 2022-07-14

**Authors:** Christopher J. Sweeney, Melanie Bottoms, Sian Ellis, Gregor Ernst, Stefan Kimmel, Stefania Loutseti, Agnes Schimera, Leticia Scopel Camargo Carniel, Amanda Sharples, Frank Staab, Michael T. Marx

**Affiliations:** ^1^ Syngenta, Jealott's Hill International Research Centre Bracknell Bracknell Berkshire UK; ^2^ Corteva Agriscience Abingdon Oxfordshire UK; ^3^ Bayer, CropScience Division Monheim Germany; ^4^ Corteva Agriscience Munich Germany; ^5^ ADAMA Deutschland Cologne Germany; ^6^ BASF São Paulo Brazil; ^7^ FMC Philadelphia Pennsylvania USA; ^8^ BASF Limburgerhof Germany

**Keywords:** Arbuscular mycorrhizal fungi, Soil ecotoxicology, Ecological risk assessment, Pesticides

## Abstract

Arbuscular mycorrhizal fungi (AMF) perform key soil ecosystem services and, because of their symbiotic relationship with plant roots, may be exposed to the plant protection products (PPPs) applied to soils and crops. In 2017, the European Food Safety Authority (EFSA) released a scientific opinion addressing the state of the science on risk assessment of PPPs for in‐soil organisms, recommending the inclusion of AMF ecotoxicological testing in the PPP regulatory process. However, it is not clear how this can be implemented in a tiered, robust, and ecologically relevant manner. Through a critical review of current literature, we examine the recommendations made within the EFSA report and the methodologies available to integrate AMF into the PPP risk assessment and provide perspective and commentary on their agronomic and ecological relevance. We conclude that considerable research questions remain to be addressed prior to the inclusion of AMF into the in‐soil organism risk assessment, many of which stem from the unique challenges associated with including an obligate symbiont within the PPP risk assessment. Finally, we highlight critical knowledge gaps and the further research required to enable development of relevant, reliable, and robust scientific tests alongside pragmatic and scientifically sound guidance to ensure that any future risk‐assessment paradigm is adequately protective of the ecosystem services it aims to preserve. *Environ Toxicol Chem* 2022;41:1808–1823. © 2022 The Authors. *Environmental Toxicology and Chemistry* published by Wiley Periodicals LLC on behalf of SETAC.

## INTRODUCTION

Arbuscular mycorrhizal fungi (AMF) are key members of the soil ecosystem, forming associations with an estimated 200,000 plant species (van der Heijden et al., 2015). As obligate symbionts, they provide their plant partners with nutrients in exchange for photosynthetically derived carbon. This symbiosis can lead to increased crop nutritional status and quality (Begum et al., 2019); however, may not always be beneficial (Miozzi et al., 2019), and the relationship between plant and AMF may fall on a continuum between mutualism and parasitism (Purin & Rillig, 2008). Arbuscular mycorrhizal fungi perform critical ecosystem services (Gianinazzi et al., 2010), for example, by contributing to improved soil structure (Rillig & Mummey, 2006) and carbon sequestration (Wilson et al., 2009), while also providing plants with protection from drought, pests, and other external abiotic stressors (Begum et al., 2019). In agroecosystems, AMF may account for approximately one‐third of soil microbial biomass (Olsson et al., 1999) and, because of their close intergrowth with plants, may be exposed to the plant protection products (PPPs) applied to soils and crops (Hage‐Ahmed et al., 2019). This exposure may be at very different developmental stages during asymbiotic or symbiotic life phases (Hage‐Ahmed et al., 2019). For example, PPPs may influence AMF development during spore germination (Mallmann et al., 2018) and initial hyphal elongation (Giovannetti et al., 2006) or the development of symbioses (Buysens et al., 2015) and connection to the common mycorrhizal network (de Novais, Giovannetti, et al., 2019; Wipf et al., 2019). Further, mycorrhizal responses to PPP exposure are often varied in direction and magnitude depending on the compound (Giovannetti et al., 2006) or species (Malfatti et al., 2021) in question and the associated environmental context (Baumgartner et al., 2005).

Because of the potential introduction of symbiotic plant partners within the risk‐assessment process, AMF represent a unique challenge for PPP ecotoxicology. Currently, no obligate symbionts are included within the PPP risk assessment. Accordingly, it is essential that, prior to the introduction of the regulatory assessment of the effects of PPPs on AMF, a reliable, robust, and validated suite of tests is established on which a tiered and ecologically relevant risk assessment process can be based. To date, there is no regulatory framework requiring the testing of PPPs on AMF. In 2017, the European Food Safety Authority (EFSA) released an extensive commissioned document (Ockleford et al., 2017), hereafter termed the *EFSA Soil Opinion*, describing the need for the introduction of a new suite of tests that incorporate mycorrhiza into the current PPP regulatory paradigm. However, the particulars of such an assessment remain uncertain. The EFSA Soil Opinion discusses both AMF and ecto‐mycorrhizal fungi in the context of ecosystem service provision, but only AMF are included in the overall recommendations. Therefore, the goal of this review will be to evaluate the feasibility of adding AMF to the PPP risk assessment using current methods, the ecological relevance of these tests, and the level of protection this may provide to AMF ecosystem service provisioning. To do this, we will provide opinion and commentary on how AMF may be integrated into a tiered risk‐assessment framework (Rohr et al., 2016). Our overall goal is to identify key data and knowledge gaps around the methods available to study AMF ecotoxicology. We will begin with a discussion of potential first‐tier in vitro studies before expanding to possible intermediate‐tier approaches and a discussion of the field studies and the assessment of AMF at the community level.

Throughout this review, we will draw on some examples involving historical PPPs that are no longer registered in many regions of the world. Despite this, they remain valid examples of AMF ecotoxicology because we can still use studies investigating these compounds to examine the reliability, robustness, and validity of the methods used. This limitation highlights the overall lack of research into AMF ecotoxicological risk assessments and the need for more studies assessing the impacts of PPPs on AMF and for such studies to use and explore methods appropriate for the generation of ecotoxicological endpoints, such as dose–response designs and establishment of toxic reference tests and suitable concentration gradients representative of field‐use scenarios.

## TIER 1: METHODOLOGICAL APPROACHES

In vitro methods are commonly used as the first tier of ecotoxicological risk assessment (Ockleford et al., 2017). Tier 1 tests are an important component of the risk‐assessment process. Tests at this level generally offer a low level of ecological relevance, maximal exposure to a test substance, and little chance for compound degradation throughout the test period. Depending on the respective specific protection goals in a tiered risk‐assessment approach, Tier 1 tests can be useful tools to filter out harmless substances that do not require further investigation under more environmentally realistic exposure scenarios in higher‐tier testing frameworks. Several in vitro endpoints have been used to assess the effects of PPPs on AMF, representing both asymbiotic and symbiotic stages of AMF development, which could be potential Tier 1 test methods in a future AMF‐PPP risk assessment. These include spore germination (Malfatti et al., 2021; Mallmann et al., 2018), hyphal length (Giovannetti et al., 2006), mycorrhization (Campagnac et al., 2008), anastomoses (de Novais, Giovannetti, et al., 2019), and spore production (Buysens et al., 2015). However, between the available methods, there is considerable variability in their reliability, reproducibility, and sensitivity (Hage‐Ahmed et al., 2019) that affect whether they are suitable candidates for inclusion into a future AMF risk assessment. The merits of in vitro approaches for studying AMF ecotoxicology will now be discussed.

Because of the inability to axenically culture AMF (Fortin et al., 2002), many studies choose to use symbiotic endpoints to assess how PPPs affect mycorrhizae (Wan et al., 1998; Zocco et al., 2008). Symbiotic endpoints using in vitro methods require the use of root organ cultures to allow symbioses to develop within a Petri dish (Fortin et al., 2002). These tests often use “hairy root” cultures formed via the transformation of root tissues with root‐inducing transfer DNA (Ri T‐DNA; Calonne et al., 2010; Wan et al., 1998). In these studies, continuous in vitro root production can be achieved, enabling mycorrhizal colonization, without the need for aboveground plant tissues (Bécard & Fortin, 1988; Danesh et al., 2006; Srinivasan et al., 2014). Wan et al. (1998) used Ri T‐DNA–transformed *Daucus carota* roots associated with *Glomus intraradices* (now *Rhizophagus intraradices* [Walker et al., 2021]) to show that this system could be used to detect dose responses of reductions in percentage of root colonization following exposure to increasing concentrations of copper sulfate, glyphosate, and α‐amino‐3‐hydroxy‐5‐methyl‐4‐isoxazole propionic acid, with no effects observed for benomyl, chlorothalonil, and dimethoate. Both Zocco et al. (2008) and Campagnac et al. (2008) examined mycorrhization of *D. carota* by *R. intraradices* following exposure to the fungicides fenpropimorph and fenhexamid, with Zocco et al. (2008) reporting no effects and Campagnac et al. (2008) reporting significant declines driven by fenpropimorph only. Note, these above were conducted in a growth medium, for example, agar or modified Strullu–Romand (MSR) medium. The authors suggest that the use of growth medium limits the comparability of outcomes to field effects and dosages attributable to the lack of influence of soil physical and chemical properties (Wan et al., 1998; Zocco et al., 2008). Consequently, a soil medium may be preferable to increase the ability to read‐across from lower‐tier to higher‐tier methods. Of course, an artificial medium provides a worst‐case exposure scenario, so the use of soil as a test medium could also be considered a refinement approach to enhance the realism of the test system as and when required.

Root organ culture methods have been extended to record other symbiotic endpoints including spore production, hyphal growth, and rates of anastomoses (Calonne et al., 2010; Campagnac et al., 2008; Cardenas‐Flores et al., 2011; Zocco et al., 2008). However, spore production in control treatments may exhibit considerable variability (Zocco et al., 2008), reducing the ability to detect PPP‐induced responses. For example, Zocco et al. (2008) could not detect statistical differences in spore production of *R. intraradices* following exposure to fenpropimorph despite the spores declining from approximately 1000 in the control to <200 in the first treatment level. Variability issues could be addressed via alterations of the experimental design to accommodate increased replication. However, further research would be required to determine the number of replicates required to detect differences between treatments at an appropriate resolution. It remains unclear if spore production endpoints could provide the statistical power to detect treatment effects within a reasonably sized test system. Meanwhile, there is a shortage of studies examining the effects of PPPs on mycorrhizal anastomoses in vitro (Cardenas‐Flores et al., 2011), preventing a complete assessment of its use. Finally, although extraradical hyphal length has been measured as a symbiotic endpoint by a number of studies using root organ culture methods (Calonne et al., 2010; Gong et al., 2014), asymbiotic methods to produce this metric (Chiocchio et al., 2000; Giovannetti et al., 2006; Pasaribu et al., 2011; Zocco et al., 2008) would likely be preferable, to reduce the complexity and variability of the test system because there is no requirement for the presence of a plant or plant tissues. Further, it has been argued that the asymbiotic measures of germinative hyphal growth represent a more sensitive endpoint because of the vulnerability of the hyphae during initial hyphal elongation following germination (Hage‐Ahmed et al., 2019; Logi et al., 1998).

Although there has been some success in the use of in vitro symbiotic endpoints to assess PPP effects on AMF, overall, there are limitations associated with their use. Many studies have shown that the cultured root tissues themselves are directly affected by PPP exposure as measured by reductions in biomass (Campagnac et al., 2008; Gong et al., 2014; Wan et al., 1998; Zocco et al., 2008). The ubiquity of these responses across multiple compounds (e.g., chlorothalonil, carbendazim, thiram, fenpropimorph, and fenhexamid), and not limited to herbicides but also including fungicides, suggests that these responses may be universal and that root organ cultures may be particularly sensitive to PPP exposure above and beyond that of a whole‐plant system. It may be possible to reduce these effects through the separate compartmentalization of root and hyphal tissues to limit exposure of plant tissues to PPPs (Hage‐Ahmed et al., 2019). For instance, Zocco et al. (2008) found that the cultured roots of *D. carota* grown on fenpropimorph or fenhexamid amended MSR medium exhibited significant phytotoxic responses including reduced biomass, yellowing, and inhibition of secondary root growth. However, these effects were completely alleviated in a bicompartmental system in which Zocco et al., 2008, cultured roots on a PPP‐free medium and only the extraradical hyphae were exposed to fenpropimorph or fenhexamid amended MSR medium. Despite this, it remains challenging to disentangle the direct and indirect effects of PPPs on endpoints, such as mycorrhization, spore production, and rates of anastomoses, because one could not easily rule out indirect effects mediated via effects on root tissue health. Tier 1 tests are designed to be relatively simple test systems which provide easily interpretable data. We suggest that the compartmentalization of root and extraradical hyphae adds significant complexity to a test design and, therefore, does not meet the requirements for a basic Tier 1 test. This suggests the need to focus on asymbiotic endpoints in the lower‐tier assessment and leave the more complex symbiotic interactions as a higher‐tier or refinement option when increasingly complex and more realistic study designs are necessary.

Asymbiotic in vitro endpoints are primarily focused on spore germination and germinative hyphal growth, and comparatively more studies have examined the effects of PPPs on AMF in the absence of symbiosis. Many studies use methods similar to the existing ISO 10832 standard (International Organization for Standardization [ISO], 2009) for examining the effects of pollutants on mycorrhizal fungi (Giovannetti et al., 2006; Malfatti et al., 2021; Mallmann et al., 2018). Here, a “spore sandwich” is created by their placement within a pair of nitrocellulose membranes which are positioned within a PPP‐treated soil substrate, allowing for the retrieval and assessment of spore and hyphae status at the end of the test period. Alternatively, spores can be placed directly on a PPP amended medium (e.g., on MSR medium) and monitored (Buysens et al., 2015; Calonne et al., 2010; Chiocchio et al., 2000; Zocco et al., 2008), although, as previously suggested, this represents a worst‐case exposure to PPPs, with a soil medium likely offering a more environmentally realistic exposure scenario. Asymbiotic methods have been demonstrated to be able to detect suitable dose–response effects, when present (Malfatti et al., 2021; Mallmann et al., 2018). For instance, Chiocchio et al. (2000) reported dose‐dependent reductions in germinative hyphal growth and spore germination of *Glomus mosseae* (now *Funneliformis mosseae*) following benomyl exposure on treated agar. Calonne et al. (2010) show similar responses of *Glomus irregulare* (now *Rhizophagus irregularis* [Formey et al., 2012]) spore germination on MSR media in the presence of propiconazole. Likewise, Mallmann et al. (2018) found increasing doses of chlorothalonil, mancozeb, and metalaxyl‐M to reduce spore germination of *Gigaspora albida* and *Rhizophagus clarus* on an artificial soil medium. Conversely, Malfatti et al. (2021) reported low levels of herbicide toxicity on spore germination of *R. clarus* and *Gigaspora albida* after exposure to glyphosate or a diuron and paraquat mixture in artificial soil and only with applications significantly exceeding the predicted environmental concentrations. These studies may, however, suffer from significant variability, even when using the ISO 10832 mandated species. For example, variability in spore germination in Giovannetti et al. (2006) across control studies for *F. mosseae* ranges from 42% to 88%, limiting the assessment of proper dose responses and highlighting potential issues with variability and reproducibility. Overall, however, spore germination and germinative hyphal length measures derived using these methods are generally similar, with similar dose–response effects being reported (Chiocchio et al., 2000; Zocco et al., 2008). This suggests that these studies have the ability to detect potentially detrimental effects on AMF that require further investigation through higher‐tier testing. Furthermore, both spore germination and germinative hyphal length measures appear to be in agreement when no effects of PPP exposure are present (Pasaribu et al., 2011; Tommerup & Briggs, 1981). For example, Pasaribu et al. (2011) found the herbicides glyphosate and alachlor to have no effect on spore germination or germinative hyphal growth of *Glomus mosseae* at up to 10 times field application rates in a soil medium. However, many studies do not include a toxic reference treatment; therefore, it is difficult to establish the true sensitivity of the test system in these cases.

Overall, the evidence suggests that the in vitro assessment of the asymbiotic endpoints spore germination and/or germinative hyphal length is a more robust and reliable alternative to symbiotic metrics and may be suitably discriminatory to be utilized as a first tier of ecotoxicological risk assessment to test the impacts of PPPs on AMF. Because the two endpoints perform similarly, it is important to establish which endpoint should be used within a risk‐assessment framework with a focus on reliability, reproducibility, and sensitivity; but this is unclear from the literature. It should be expected that endpoint sensitivity differs between PPPs; however, comparison of the endpoints is challenging because studies may not have reported effective concentration (EC*x*) values or provided statistical assessment of the results (Giovannetti et al., 2006), methodologies may not be suitable to establish a complete dose response (Schreiner & Bethlenfalvay, 1997), or studies simply reach different conclusions. For example, Zocco et al. (2008) found that the half‐maximal inhibitory concentrations (IC50s) of spore germination of *R. intraradices* in MSR medium were 6.1 and 5.6 mg L^−1^, respectively, for fenpropimorph and fenhexamid exposure but 6.4 and 9.3 mg L^−1^, respectively, for germinative hyphal growth, although the four‐step, 1000‐fold concentration range is better suited to a range‐finding test than the calculation of accurate endpoints. Conversely, other studies indicate that germinative hyphal growth may be the more sensitive parameter (Chiocchio et al., 2000; Giovannetti et al., 2006). However, spore germination, as a percentage metric, is bounded by the number of spores used within the test. This provides an advantage over the continuous measure of hyphal growth, which can both increase and decrease relative to a control treatment. For instance, in the study of Giovannetti et al. (2006) the fungicide metalaxyl appeared to have no effect on *Glomus mosseae* spore germination, whereas germinative hyphal growth doubled at the highest application rate relative to the control. The interpretability of an increase in germinative hyphal growth is challenging from an ecotoxicological perspective because it is not clear whether this should be considered as an adverse response to the PPP in question. For example, increased rates of extraradical hyphal growth are considered ecologically beneficial in response to plant‐derived compounds, such as those found within root exudates (Buee et al., 2000; Nagata et al., 2016; Tsai & Phillips, 1991). Conversely, in a spore germination test a negative response is demonstrably clear in all scenarios.

Overall, the suggestion in the EFSA Soil Opinion of further exploring the use of an AMF spore germination test within the battery of tests used in the PPP risk assessment appears to be a sensible approach. Spore germination studies would likely support the proposed protection goals outlined in the EFSA Soil Opinion in which AMF biomass and abundance are considered to be the units of preservation because AMF spores are a vital component of AMF survival and the maintenance of potential soil infectivity during the winter and fallow periods (Jamiołkowska et al., 2018; Kabir, 2005). However, this assumes that spore germination equates to soil mycorrhizal infectivity, but the relationship between laboratory tests and field performance is not yet understood. It is also clear that with our current understanding the available test systems are not at a stage in which reliable and reproducible dose–response outcomes can consistently be derived. While Tier 1 test systems by their very nature are artificial and limited in their real‐world relevance, addressing key concerns and research needs, which will now be discussed, is critical to optimizing the ecological and agronomic relevance of the test system.

## TIER 2: UNKNOWNS OF IN VITRO AMF SPORE GERMINATION TESTS

While the asymbiotic in vitro testing of AMF spore germination should be considered for future Tier 1 risk‐assessment purposes, significant knowledge gaps remain that need to be addressed to allow for a consistent and reliable risk‐assessment process. The first and, possibly, most important issue is to develop our understanding of how test outcomes should be interpreted. Some spores that do not germinate following exposure to PPPs within the test period can subsequently germinate when placed on a PPP‐free medium (Buysens et al., 2015; Chiocchio et al., 2000; Zocco et al., 2008). This suggests that a proportion of PPPs may be fungistatic rather than fungicidal and may inhibit germination rather than kill the spore. It does not make ecological sense to consider an ungerminated but viable spore in the same manner as a dead spore. This means that recovery from PPP exposure may be possible, particularly as a given PPP degrades in the environment over time and falls under a threshold level under field conditions. Unfortunately, the ISO 10832 interlaboratory comparison showed field soils performing poorly in terms of reproducibility, with artificial soil producing mostly consistently high levels of spore germination across control treatments (ISO, 2009). The use of artificial substrates in combination with the short 14‐day test period would greatly limit the potential for PPP degradation during the test period, and it is therefore reasonable to assume that the recovery of spore germination would not be captured within the current ISO 10832 methods. We recommend that further research considers how to integrate the viability of ungerminated spores at the end of a test period and their subsequent germination into the risk‐assessment process. This recovery potential could be considered as a potential intermediate‐tier or refinement approach to develop the risk assessment beyond the first tier. For instance, it may be possible to extend test periods and replace the PPP‐treated medium with one of a reduced concentration as informed by appropriate models to imitate PPP degradation or in combination with an aged residue‐style study. This would be important information to enable risk assessors to fully comprehend the effects of a given PPP on AMF, particularly because the ability of a population to recover is fundamental to the proposed protection goals outlined in the EFSA Soil Opinion. Overall, this represents a key knowledge gap because we do not fully understand the potential for fungistatic impacts of PPPs on AMF spores and the subsequent recovery of spore germination over time. We encourage researchers to incorporate spore viability testing into future PPP AMF spore germination tests. This could be achieved by utilizing tetrazolium salt tests to detect actively respiring spores (Druille et al., 2013; Stentelaire et al., 2001) or, preferably, by transferring spores to clean media and monitoring for further germination (Buysens et al., 2015; Chiocchio et al., 2000; Zocco et al., 2008). Further data generation will enable the assessment of whether the ISO standardized testing methodologies need adapting to incorporate fungistatic test outcomes.

Arbuscular mycorrhizal fungi represent a hugely diverse clade of fungi (Schüβler et al., 2001); however, considerable intraspecific genetic diversity exists within species (Mathieu et al., 2018; Munkvold et al., 2004). For example, Chen et al. (2018) describe significant variability in the genomes of five isolates of *R. irregularis* sampled from the same field. Isolate‐level variation could be a key source of variability between studies and laboratories conducting ecotoxicological experiments. However, few studies have assessed how this can affect the outcome of ecotoxicological tests on AMF. de Novais, Giovannetti, et al. (2019) examined asymbiotic measures of germinative hyphal length and mycelium viability across three *F. mosseae* isolates using the “spore sandwich” method described in the previous section. Germinative hyphal length of some isolates was unaffected by PPP exposure (up to twice the field application rate), whereas others exhibited significant dose–response declines. For instance, following exposure to dicamba and benomyl, *F. mosseae* IMA1 showed reduced hyphal growth, but *F. mosseae* BEG119 and BEG116 were unaffected. In contrast, germinative hyphal length of *F. mosseae* BEG119, but not BEG116 or IMA1, was reduced following exposure to fenhexamid (de Novais, Giovannetti, et al., 2019). To our knowledge, no studies have assessed the isolate‐level variability in the outcome of PPP ecotoxicological tests on spore germination.

The ISO 10832 guidelines mandate the use of the BEG12 isolate of *F. mosseae*. Given the discussed variability in isolate‐level AMF responses to PPPs, it would be beneficial to explore if this variability is also present within spore germination responses. This would be highly relevant research for the long‐term applicability of the test guidelines because AMF spore viability declines over time (Mallmann, 2020), meaning that fresh spores will need to be continually produced in testing laboratories via the long‐term culture of AMF isolates. However, because of the multinucleate nature of AMF (Kokkoris et al., 2020), significantly altered phenotypes can arise from the prolonged culture of a single AMF isolate (Ehinger et al., 2012; Kokkoris & Hart, 2019; Sbrana et al., 2018). Wyss and Bonfante (1993) propagated samples of an isolate of the ISO mandated *F. mosseae*, which had been cultured for 12 years across different laboratories, revealing genotypic divergence among the different lineages. It is currently unknown how this genetic divergence could introduce variability into an ecotoxicological test system. An understanding of the intrinsic intraspecific variability in the response of AMF spore germination to PPP exposure would be beneficial to assess the significance of this potential genetic divergence over time. It would be unwise not to examine this because genetic divergence between lineages derived from a single isolate present within different testing laboratories could introduce unwanted stochasticity into the risk assessment. We note that there are many isolates or species across different AMF families and ecotypes that could be potentially selected for use in a risk assessment outside of the ISO mandated *F. mosseae* BEG12 isolate. Importantly, the issue of intraspecific diversity and genetic divergence between laboratories would be relevant for any isolate selected. Therefore, a toxic reference test using different isolates could be key to determine sufficiently sensitive isolates for reliable and replicable ecotoxicological testing.

The issue of isolate variability extends beyond the outcome of the ecotoxicological tests and affects the methodologies. Mallmann et al. (2018) suggest that incubation temperatures during spore germination tests will need to be altered depending on the global region from which the isolate is produced. In their case an increase in incubation temperature to 28 °C from the ISO 10832 recommended 24 °C was necessary to satisfy validity criteria and enable sufficient germination from subtropical and tropical AMF isolates (Mallmann et al., 2018). In a wider ecological context, it is known that isolates of the same AMF species from varying climatic zones may undergo ecotypic differentiation (Antunes et al., 2011). Any future guidelines used to inform AMF ecotoxicological tests may wish to consider being able to accommodate methodological variability based on the needs of spores isolated from contrasting global regions, without the risk of registrants being penalized for deviations from standardized methods.

Methodological variability is also an issue for interspecific variation within AMF testing. It would be easy to argue that to accommodate unknown variability within the risk‐assessment process, additional AMF species should be included in the battery of tests to be conducted; however, this is not a simple solution and highlights considerable methodological complexities within the test system. The ISO 10832 guidelines only provide methodologies for *F. mosseae*. Mallmann et al. (2018) and Malfatti et al. (2021) build on this, showing that both *Gigaspora albida* and *R. clarus* are suitable candidates for use and can satisfactorily meet the validity criteria within the ISO framework. However, for these species boric acid (which is used or has been proposed as a toxic reference substance for tests on other soil organisms [Niemeyer et al., 2018]), rather than the ISO 10832 recommended cadmium nitrate, was required to act as an appropriate toxic reference substance (Mallmann et al., 2018). Indeed, no IC50 values for cadmium nitrate could be derived by Mallmann et al. (2018) despite the use of concentrations approximately 10‐fold larger than those used in the ISO methodologies. In addition, three of the five species tested by Mallmann et al. (2018) failed to satisfy validity criteria because of insufficient germination and spore recovery, meaning that not all AMF species will be suitable for use within these tests. This highlights the interspecies variability within spore germination tests. It must be acknowledged that, for any species tested, it may be required that variations in protocols are needed to maximize spore germination percentages and satisfy appropriate validity criteria. This may take the form of altered incubation temperatures, the use of alternate toxic reference substances, or even variation in the time period of the experiment. For instance, the ISO 10832 test period is 14 days long, but Mallmann et al. (2018) show that through a combination of different species and their need for different incubation temperatures an extension of the test to 28 days could improve germination in some cases. In addition, it may be required to consider the use of different media between species because Mallmann et al. (2018) report species‐level variations in spore germination based on the use of sand, tropical artificial soil, or Organisation for Economic Co‐operation and Development artificial soil. Furthermore, different validity criteria may need to be developed for each species tested. Together, the variability in conditions required to optimize germination for any given species or isolate may mean that a single universal methodology of AMF spore germination testing may not be possible across all species and all PPPs, a conclusion also reached by Mallmann et al. (2018). This is not compatible with current highly standardized lower‐tier methodologies in the soil organism risk assessment and may limit the ability to include AMF within the risk‐assessment framework. Overall, the complexity introduced by testing multiple species at Tier 1 may mean that further species should only be considered for inclusion in the risk assessment as a higher‐tier approach, should it be required and following appropriate ring testing and validation.

To be protective, the risk‐assessment process must try and represent the diversity of soil organisms; however, it is impossible to test all compounds on all AMF taxa. The EFSA Soil Opinion suggests that for soil invertebrates an appropriate and calibrated assessment factor can cover the intra‐ and interspecific variability in toxicological sensitivity between species. However, to date, no such assessment factor exists or has been suggested for AMF endpoints. The goal of this assessment factor would be to address uncertainties when extrapolating laboratory tests to infer what would happen if mesocosm or field studies were conducted. This would be the result of an appropriate calibration process assessing the differences between lower‐ and higher‐tier studies and how protective these tests are of a suite of protection goals. Such a calibration may not be possible in the context of AMF spore germination tests because this metric would not be possible to measure under field conditions because methods do not exist to monitor spore germination within PPP‐treated field soils. Likewise, it is not currently known how in vitro measures of spore germination relate to the field performance of AMF populations. Furthermore, it is still unclear which AMF parameters will be the targets of preservation of the protection goals for the field risk assessment of PPPs, that is, structural versus functional endpoints of AMF, aside from the difficulties of measuring these under realistic field conditions. Consequently, it would be challenging to calibrate this metric. The EFSA Soil Opinion notes that current assessment factors within the soil organism risk assessment do not have a transparent rationale. Given the continued debate in better‐established areas of the soil organism risk assessment (Christl et al., 2016), we strongly encourage further discussions and research as to how to handle uncertainty within a potential future AMF risk‐assessment process to define an assessment factor, if one is necessary, based on sound theory, that is appropriately protective and not overly conservative.

## TIER 2: INTERMEDIATE‐ AND HIGHER‐TIER STUDIES

Higher‐tier approaches increase the environmental realism of a given study, providing more agronomically relevant assessments of the impact of PPPs on the target organism; but this, in turn, increases the complexity of the test system. Although the EFSA Soil Opinion advocates for the inclusion of mycorrhizal ecotoxicological testing within the PPP risk assessment, of note is the absence of recommendations of experimental higher‐tier approaches. It is critical that, on the introduction of AMF testing within the risk assessment, appropriate consideration is given to the necessary design and requisite statistical analyses for higher‐tier ecotoxicological data. In doing so, this will minimize uncertainty and enable the generation of data suitable for assessment and appropriate for acceptance by regulatory bodies. These higher‐tier studies should be able to generate endpoints which are relevant to currently undefined protection goals. Because there is no agreed‐on and validated design for higher‐tier studies, there is further work required to develop a regulatorily acceptable test design which can adequately detect potential effects and has acceptable levels of variability.

The work of Schreiner and Bethlenfalvay (1997) provides a thought‐provoking example of a possible intermediate‐tier testing strategy, building on the discussion of spore germination studies. They adapt the “spore sandwich” germination tests and use field soil in a potted system, revealing interesting results in the case of the exposure of *Glomus etunicatum*, *Glomus mosseae*, and *Gigaspora rosea* to benomyl and pentachloronitrobenzene (PCNB). They show in a Petri dish system with a soil medium, similar to that described in the previous sections, significant declines in almost all measures of spore germination and germinative hyphal length across all species following exposure to benomyl and PCNB. However, they expand their experimental system to bury the “spore sandwich” into sterilized soil in 600‐ml pots and apply a soil drench of the PPPs, resulting in significantly improved spore germination and germinative hyphal length compared with the Petri dish system. For example, exposure of *Glomus etunicatum* (10 mg active ingredient/kg soil) to both benomyl and PCNB in this system did not significantly reduce spore germination relative to the control. The authors assign these differences to more realistic PPP adsorptive behavior in a potted soil column. The use of sterile soil in the potted soil test system excludes the potential for microbial degradation of the compounds (Parte et al., 2017) or the interactions of AMF growth with the soil microbiome and associated growth responses (Frey‐Klett et al., 2007; Scheublin et al., 2010), meaning this remains an artificial test system. The implications of this are twofold. First, we can hypothesize that Petri dish systems to assess AMF endpoints are more conservative relative to results obtained using more realistic exposure scenarios. Second, the use of a field soil in intermediate test systems in a potted glasshouse experiment could represent a stepwise increase in complexity of the ecotoxicological risk assessment, warranting further exploration. This observation is based on one study, emphasizing the need for work to expand our understanding of how this or similar test systems may work with different soils, AMF species/isolates, and compounds. This would need to consider all of the caveats discussed in the *Tier 1: Unknowns of In Vitro AMF Spore Germination Tests* section, particularly in the light of the inconsistent germination that was reported in the ISO 10832 interlaboratory comparison of spore germination tests using natural field soils (ISO, 2009).

The study of Schreiner and Bethlenfalvay (1997) involved a potted bare soil experiment, and bare soil microcosms have also been used to study the impact of PPPs outside of spore germination tests. However, we note that the use of bare soil systems to assess endpoints relating to the symbiotic stage of the AMF life cycle lacks ecological relevance and that the absence of a plant host may detrimentally affect the mycorrhizal community independently of the PPPs applied. For instance, Rivera‐Becerril et al. (2017) used bare field soil microcosms to assess the effects of fenhexamid, folpel, and deltamethrin on AMF propagule numbers (spores, mycelium, and colonized root fragments per 100 g of dried soil sample). They note that the declines in propagule numbers they observed may not only be attributable to PPP application but also the lack of carbon inputs from a host plant. Therefore, this system is likely unsuitable for use in the risk assessment, despite the ease of incorporating PPPs into a bare soil compared with a planted system.

Potted plant experiments offer a more complex but increasingly realistic opportunity for intermediate‐tier testing and could be performed in a suitable artificial environment. Endpoints in such tests would examine the symbiotic phase of the AMF life cycle, meaning that metrics such as mycorrhization, spore production, or plant biomass could be used. Studies have successfully examined the effects of PPPs on AMF within potted systems in both glasshouse (Channabasava et al., 2015; Hernández‐Dorrego & Parés, 2010) and growth chamber (Schweiger & Jakobsen, 1998) environments. These methods bring with them significant challenges associated with the testing of herbicides which may kill the plant host but could be suitable for the testing of insecticides and fungicides. A potted plant test system could also be appropriate for the testing of fungicidal or insecticidal seed treatments because herbicides are not used in this way. Cameron et al. (2017) tested the effects of several commonly sold fungicidal seed coatings on the mycorrhization of corn, soybean, and oats in a glasshouse experiment, reporting no significant effects. In a similar glasshouse experiment, Burrows and Ahmed (2007) tested the effects of seed coatings on the AMF colonization of muskmelon, squash, bean, tomato, and corn and describe minor, inconsistent, and transient effects across plant species and sampling times. Of course, rates of AMF colonization are not necessarily directly related to the degree of AMF functioning or the benefit of the symbiosis to the plant (Füzy et al., 2015). These studies suggest that potted plant systems can be used to measure field dose‐relevant responses of AMF to PPPs, particularly in the context of seed coatings. However, this would not be applicable should such coatings be applied to non‐mycorrhizal crops, in which case alternate methods would be required.

Mesocosm‐style tests could also be used in an intermediate testing system, and studies have had success in this area. Lovatel (2017) used terrestrial model ecosystems (TMEs) to test effects of different fungicides on AMF. They found no effects on the mycorrhization of onion or AMF hyphal production and sporulation following applications of chlorothalonil, metalaxyl, or a mixture of the two. In this experiment they not only exposed the native population but introduced an AMF inoculum (*R. clarus*, *Claroideoglomus etunicatum*, *Gigaspora albida*, *Acualospora morrowiae*, *Acualospora koskei*). The inoculation increased the productivity of the onion crop independently of the fungicide applications. Mallmann (2020) also used a TME approach and tested the effects of chlorothalonil on AMF in a natural Cambisol. They found no effects of chlorothalonil on variables related to the percentage mycorrhizal colonization of soybean roots; however, the number of spores and total extraradical hyphal length declined following exposure to field‐relevant concentrations of the fungicide. These studies show the potential of the TME approach, but significant unknowns remain in how to integrate mesocosm tests into the risk assessment.

If using an unsterilized field soil medium within a mesocosm, the introduction of plants will exert an influence on the fungal community, which may take a minimum of 3 months to reach its maximum (Hannula et al., 2019). Prior to this, the fungal community will still be acclimatizing to the presence of the plants in the system. To reduce the variability within the test system, it would be beneficial to allow sufficient time for this to occur within the testing recommendations to reduce unwanted variability, which could result from differential development of the plants, and therefore their symbiotic relationships, between replicates (Chaparro et al., 2014). The introduction of a plant partner into an ecotoxicological test examining the effects of PPPs on AMF raises some additional challenges, such as the inclusion of herbicidal PPPs within ecotoxicological tests. For instance, de Novais, Avio, et al. (2019) were unable to assess the effects of the herbicide glufosinate on extraradical mycelium at field‐relevant concentrations because of limited plant survival following exposure to the herbicide. It is imperative that a risk‐assessment framework needs to accommodate testing at field‐relevant concentrations and the testing of herbicidal products. Therefore, we must consider whether we can accommodate the death of the symbiotic plant partner within the testing process and the implications of this on the study design and interpretation. We need to define appropriate model plant species for use in these tests. For broad‐spectrum herbicides, the use of any model plant species could be suitable in a mesocosm experiment. However, in the case of selective herbicides such as those targeting grasses (e.g., clodinafop‐propargyl) or broad‐leaved weeds (e.g., 2‐methyl‐4‐chlorophenoxyacetic acid), differential protocol development may be required should plant death be desired within the test system. Any such deviations reduce the standardization and increase the complexity of the risk‐assessment process.

The death of the plant will likely make endpoints such as mycorrhization impossible to measure because death will signal the end of the symbioses, and the subsequent desiccation of plant roots following herbicide treatment would make harvesting impractical. However, studies show that the extraradical mycelium may remain viable, despite the death of the symbiotic partner, although these observations were based on the removal of the aboveground plant tissues, not PPP exposure (Müller et al., 2013; Pepe et al., 2018). Therefore, measures of AMF biomass (see *Tier 3: Field Testing* section) from soil samples may remain possible because this would primarily be made up of fungal hyphae and spores external to the roots regardless of the life status of the plants in the system (Olsson et al., 1997; Olsson & Johansen, 2000). However, we do not know how reliable any measures of AMF biomass could be when a plant in the test system is exhibiting phytotoxic effects of PPP exposure. For example, there are numerous reasons why AMF could be influenced by plant death, not least the cessation of the provision of carbon to the mycorrhiza. Likewise, root systems, on plant death, also leach nutrients, particularly nitrogen and phosphorus, into the soil, which are rapidly absorbed into the mycorrhizal network (Eason & Newman, 1990; Johansen & Jensen, 1996; Müller et al., 2013). Any control treatment in such a PPP mycorrhizal mesocosm test would not be exposed to this potential nutrient flush, so we would need to understand any possible effects on AMF biomass or utilize appropriate controls within such tests, for instance, via the mechanical weeding of control treatments (Hage‐Ahmed et al., 2019). Alternatively, plant death could be incorporated into the test system by evaluating mycorrhizal interactions with a subsequent generation of plants grown on the treated soil (Hage‐Ahmed et al., 2019), potentially better reflecting the relevance of the test to the use cases of herbicidal PPPs. This is a complex issue as, on the one hand, herbicide application is intended to kill plants; therefore, one could argue that the death of the plant is representative of realistic field‐use scenarios. However, society also accepts the use of herbicides to remove weeds; therefore, one could also propose that indirect effects on AMF and the end of symbiosis via the death of the weed are indicative of the use case of the PPP, and therefore must also be acceptable. Mesocosm tests may be possible within the context of AMF ecotoxicological risk assessment, but we require a deeper understanding of the indirect effects of PPPs on AMF because of effects mediated via plants within the test system.

## TIER 3: FIELD TESTING

For some compounds, assessment may be required at the highest level of ecotoxicological testing within the risk assessment: field studies. Several field studies have assessed the impacts of PPPs on AMF communities, with positive, neutral, and negative effects of varying magnitudes reported. For example, reductions in AMF diversity were observed following oxyfluorfen application, at a rate above the allowed annual field rate, in Mediterranean soils (Alguacil et al., 2014); yet diversity remained unaffected following long‐term use of flumioxazin and paraquat in American peach orchards (Zhang et al., 2018). Carrenho et al. (1998) examined soils of citrus farms and found that reductions in AMF diversity as a result of fosetyl‐al were site‐dependent, while low doses of metalaxyl increased spore diversity. Meanwhile, the negative effects of carbendazim on AMF community structure and mycorrhizal colonization were found to be transient over a 90‐day field experiment (Ipsilantis et al., 2012). Similar transient effects on mycorrhizal colonization following fluazifop‐*p*‐butyl and fomesafen applications on bean plants have been reported by Santos et al. (2006), with declines observed 12 days after treatment but subsequently recovering over a 51‐day observation period. Other contrasting field observations have been made for AMF colonization following PPP exposure in the field, including no effects following chlorothalonil, fenarimol, and iprodione applications to a golf green over 6 months (Bary et al., 2005); an average reduction of 53% following flazasulfuron, glufosinate, or glyphosate within‐row applications in a vineyard compared to mechanical weeding (Zaller et al., 2018); and no effects following application of 4‐(2‐methyl‐4‐chlorophenoxy)butyric acid in a pea cropping system in which tillage was the primary factor determining colonization rates (Rosner et al., 2020). We must also be aware of interacting factors that can affect AMF. For instance, mycorrhization of grape vines was shown to depend on interactions between seasonality and weed control method (Baumgartner et al., 2005). Meanwhile, nicosulfuron applications (following an atrazine pretreatment) in a corn crop at field doses were found to increase AMF spore number and total soil glomalin content in a study by de Freitas et al. (2018).

Despite a multitude of studies examining PPP effects on AMF in the field, very few employ a field‐realistic dose‐related study design, meaning that extrapolation of the findings to a risk assessment context is challenging. It is important that any results can be contextualized by the intended use cases of the compounds in question, to ensure agronomic relevance. The study of Jakobsen et al. (2021) tackles this, showing that rates up to 1 times the field dose of mancozeb increase mycorrhizal colonization in peas, which subsequently declined at 5 times and 25 times application rates. However, the range of concentrations used in that study is too wide to derive ecotoxicological endpoints. Karpouzas et al. (2014) employed a more realistic dosage in their field experiment, exposing maize to 1, 2, and 5 times the field rate of nicosulfuron and reporting no significant effects on mycorrhizal colonization or AMF community structure. Further studies that employ field‐realistic dose‐related designs within appropriate concentration ranges are critical to enable an understanding of how mycorrhizal endpoints may be generated within a higher‐tier ecotoxicological study.

Field studies have the advantage that test conditions are invariably designed to emulate real‐world use scenarios of the PPP in question. Therefore, the monitoring of AMF interactions with crops grown in the soil in which an herbicidal product has been used offers significant potential for ecological and agronomic relevance of the test system. The measurement of AMF biomass within the soil could represent a suitable endpoint for use in field studies, particularly by employing fatty acid analysis as an indirect measurement of biomass (Olsson & Lekberg, 2022; Vestberg et al., 2012). Neutral lipid fatty acids (NLFAs), and in particular NLFA 16:1ω5 (an AMF storage lipid [Lekberg et al., 2013]), have been shown to be sensitive biomarkers for AMF quantification (Sharma & Buyer, 2015). Existing methods within ISO 29843‐2:2011 should accommodate such analyses within standardized frameworks. Sharma and Buyer (2015) found that NLFA 16:1ω5 was positively related to AMF spore density. Vestberg et al. (2012) report NLFA 16:1ω5 to be highly correlated with the most probable number method of estimating AMF propagule numbers, linking NLFAs quantitively to soil AMF infectivity. Also, NLFA 16:1ω5 has been used as an indicator of hyphal length density (Ven et al., 2020) and root colonization by AMF (Barceló et al., 2020), meaning it has wide ecological relevance. Fatty acids are rapidly decomposed in the soil, with a short half‐life, making them a useful indicator of live biomass and suitable for use in ecotoxicological experiments (Zhang et al., 2019) in which discrimination between live and dead tissues would be essential. Neutral lipid fatty acids have been used to measure the impacts of PPPs on AMF. For example, Zaller et al. (2014) show reductions in NLFA 16:1ω5 following glyphosate exposure. Furthermore, NLFA analysis can discriminate between soils cropped with mycorrhizal or nonmycorrhizal plant species, supporting its ability to detect real‐world differences within the AMF community (Vestberg et al., 2012). We are unaware of any study utilizing NLFAs to generate ecotoxicological endpoints. It would be of great relevance to establish an experiment in which increasing dosages of PPPs are applied to determine whether NLFA analysis could be used to detect dose‐dependent effects of PPPs on the mycorrhizal community. This should be done from the bottom up by testing the validity of the approach from the laboratory through to the field and assessing whether this method is suitable for use in cases where indirect effects on AMF can be anticipated, such as with herbicidal effects on a plant symbiont. This would enable us to understand the sensitivity of the method and the timescales for effects and subsequent recovery of the AMF population following PPP exposure. Likewise, we need to understand the variability and reliability of NLFA analysis and the response of the metric to PPP applications in different soil types, regions, and AMF communities of differing composition.

Ecotoxicological field studies of AMF present many important challenges relating to validity and realism. These challenges are shared with other soil organism field studies and are addressed by regulatory guidance, such as that for earthworm field studies, so we may look to these studies to consider what may be deemed a valid field trial by regulators for AMF exposure to PPPs. Regarding validity, the current earthworm field guidance recommends the presence of a prescriptive number of individuals per land area and that ecologically important species for a given environment must be present above threshold abundances (ISO, 2014). It is unclear how such validity criteria could be implemented for AMF because we currently lack an understanding of the natural variability in field communities and how this could influence the risk assessment. Hannula et al. (2021) illustrate this variability, reporting that just 3% of AMF operational taxonomic units, identified during the sequencing of fungal community composition across 12 European long‐term agricultural experiments, were shared between three or more countries. Similarly, Rivera‐Becerril et al. (2017) report that each field soil they tested possessed its own AMF community. This between‐field variability could mean it may not be possible to mandate that specific ecologically relevant species must be present for the test to be valid, as with other existing frameworks. We also do not currently fully understand which AMF species are considered more ecologically or functionally important. This variability is not just limited to diversity but may also encompass AMF abundance. Although validity criteria are set for the abundance of earthworms required in ecotoxicological field testing, we lack an understanding as to whether it could be possible to mandate a specific abundance of AMF in a field used for PPP risk assessment. To the best of our knowledge, no study has provided an estimate of the “average” AMF biomass in certain soils, likely because these metrics vary significantly with soil properties, which change across various spatial scales (Hannula et al., 2021; Schlatter et al., 2018). However, a range of potential biomass values necessary for a valid trial is conceivable if backed by sufficient ecological data. Consequently, based on current knowledge, it may not be possible to define validity criteria for potential AMF ecotoxicological field tests, at least as far as they are currently implemented within the PPP risk‐assessment process.

One way to address the effects of variability between studies on AMF in the field would be to use a toxic reference substance, as is done in current earthworm PPP field studies (ISO, 2014). In doing so, regardless of differences in AMF abundance or diversity between study sites, regulators would be able to see that the community is sufficiently sensitive to detect effects, if present. There is limited evidence as to whether a toxic reference substance for AMF could work under field scenarios, and in most studies, only effects on mycorrhization, not AMF biomass, are reported. The fungicide benomyl and its metabolite carbendazim have also been used as toxic references within AMF research, beyond their widespread use in earthworm studies. Under field conditions, carbendazim reduced AMF colonization by approximately 20% in a pea crop (Bødker et al., 2002), yet Helgason et al. (2007) applied benomyl to field soil monoliths and found that the abundance of certain AMF taxa was increased as a result of competitive release from the pressures of more sensitive species. In the study of Ipsilantis et al. (2012) carbendazim application reduced root colonization to effectively zero in a laboratory study but exerted only a minimal and transitory effect under field conditions. Meanwhile, carbendazim was also used as a toxic control under a maize cropping system but reduced root colonization only from approximately 23% to 15%, suggesting that it may be of limited use (Raya‐Hernández et al., 2020). Outside of benomyl or carbendazim, the fungicide Topsin‐M has also been suggested for use (Wilson & Williamson, 2008); however, this is likely because carbendazim is a key metabolite of Topsin‐M. Therefore, this offering likely does little other than present a more readily available compound for experimental use because benomyl is no longer produced. Wilson & Williamson (2008) found that Topsin‐M could inhibit mycorrhization of two grasses, with applications every 3 weeks over the growing seasons across a 3‐year period. However, these consistent and long‐term applications to reduce mycorrhizal activity are not suitable for regulatory ecotoxicological experiments. These studies demonstrate that we currently do not have a toxic reference substance that can be used as a positive control in AMF ecotoxicological field tests because current options clearly are not sufficient to prove the sensitivity of a test system. Further research should aim to identify such a compound. Because field tests would only be conducted once lower‐tier tests have already demonstrated some degree of toxicity for the compound, it may be possible to explore using an overdosing of the PPP being tested as a reference. However, this brings with it many risks associated with leaching and nontarget exposure, meaning that a true toxic reference would be optimal to provide a standardized reference for comparison between studies and sites.

The second topic to consider for the field testing of PPPs on AMF is realism. Within ecotoxicology methodological decisions need to be made to balance statistical power against the ecological realism of the study. For instance, in earthworm field testing (ISO, 2014) grassland soil is commonly used as a surrogate for cereal crops because of an increased abundance and diversity of earthworms compared with arable soils. Therefore, we should consider whether such trade‐offs are appropriate in the context of field site selection for AMF PPP field testing. As already discussed in this section, there is considerable variability in AMF diversity within agricultural arable fields; however, considerably larger differences exist between cropped and noncropped soils (Oehl et al., 2003; Wang et al., 2015). This is in contrast to earthworms in which the same species can be commonly found within grassland or arable soils (Satchell, 1983). Grassland AMF diversity and abundance are considerably higher than in arable soils (Oehl et al., 2004), although agricultural soils may accommodate surprising amounts of AMF diversity (Baltruschat et al., 2019). It has been shown that agricultural soils likely harbor a more resilient and ruderal AMF community of very different composition to more natural systems (Chagnon et al., 2013; Öpik et al., 2006). Therefore, if the context of the risk assessment is to protect the ecosystem services that agricultural AMF provide from effects due to PPP exposure, then it stands that agricultural soils should be used in field studies. It does not seem ecologically relevant to test the effects of PPPs on AMF communities which will not be exposed to PPPs and are not best suited for representing the agricultural AMF tolerant of continual disturbances such as fertilization and tillage. Indeed, it is likely that an AMF community adapted to a non‐agricultural lifestyle would represent an enormously conservative choice, which would hold little ecological realism to the real‐world field use of PPPs. This view is supported by the EFSA Soil Opinion, which details the need for “the maintenance of biodiversity levels close to normal operating ranges for agricultural field soils.” Therefore, we consider an appropriate field test examining the impacts of PPPs on AMF to be one conducted on agricultural field soils, to ensure the representativeness of real‐world use scenarios and that tests are protective of the communities present in which the products will be used. Of course, it will remain important, as for field studies in other areas of the in‐soil organism risk assessment, that any soil meets specific selection criteria. Such criteria could include the need for a soil not to have been treated with a given or similar PPP in the years preceding a field study.

To truly consider realism in AMF studies, we also need to consider the intended use case of the products in question. There is significant variability in the mycorrhizal status of commonly cropped species from non‐mycorrhizal crops (e.g., oil seed rape, sugar beet, and mustard) to low‐mycorrhizal crops (e.g., wheat and barley) to moderately mycorrhizal crops (e.g., maize and sunflower) to highly mycorrhizal crops (e.g., carrot, potato, and pea). Cropping of non‐mycorrhizal species leads to reduced AMF abundance, diversity, and soil infectivity naturally (Bowles et al., 2017; Lekberg & Koide, 2005); therefore, we may need to consider the relevance of the mycorrhizal risk assessment to these use cases. The study of the impacts of the inclusion of non‐mycorrhizal crops within a cropping rotation could be a useful tool to compare the ecological relevance of the size of the effects of PPPs on AMF and the subsequent population recovery. This could provide additional context to the impacts of PPPs on AMF during the establishment of the risk‐assessment process and potentially within a future risk‐assessment framework. Further research and discussions among stakeholders should consider how to integrate the differential mycorrhizal dependency of crops into a risk assessment to ensure that its agronomic relevance is maximized. This is pertinent across all tiers of the risk‐assessment process and will be of continued importance as continued crop breeding and domestication lead to crops with lower nutrient dependency and reliance on mycorrhiza (Martín‐Robles et al., 2018; Weih et al., 2017; Zhu et al., 2001). Further expanding on the need for realism, one can consider other agronomic disturbances to the soil and their impacts on AMF. Tillage and fertilization can disrupt AMF hyphae and community dynamics and reduce AMF diversity and root colonization (Bowles et al., 2017; Hannula et al., 2021; Peyret‐Guzzon et al., 2016). As with non‐mycorrhizal crops, we should consider the magnitude of the effects of PPP use on AMF relative to non‐risk‐assessed agronomic practices that have strong impacts on the mycorrhizal population within agricultural soils to maintain environmental and ecological realism and aid the pragmatic interpretation of field‐generated data.

### Off‐field effects

Direct effects on AMF are not the only concern regarding mycorrhizae within the PPP risk assessment. In additional EFSA documents, examining the impact of PPPs on nontarget terrestrial plants (Aagaard et al., 2014), the authors note the potential for off‐field effects of PPP application via the mycorrhizae. Evidence suggests that off‐field areas can maintain distinct mycorrhizal communities from the cropped land (Holden et al., 2019), suggesting that the drivers of agricultural AMF communities (e.g., ploughing, fertilization) are not extending effects to non‐target areas. The ability of AMF to form anastomoses and initiate hyphal fusions is generally considered to be an intraspecies trait (Chagnon, 2014; Croll et al., 2009; Giovannetti et al., 2003). Because on‐ and off‐field communities remain distinct, it is unlikely that there will be a high frequency of hyphal fusions between these communities. Therefore, it is improbable that effects of PPPs on off‐field AMF communities will be mediated via exposure of on‐field AMF communities and the common mycorrhizal network. Any such risk may be adequately covered through the current off‐field non‐target plant risk assessment because the primary route of exposure would likely be via drift or runoff affecting nontarget plants. Under the assumption that a risk assessment is protective of the plant community in an off‐field area, we may assume that the symbionts of those plants are also protected; but this remains to be verified.

### The potential to include AMF community metrics within the risk assessment

The EFSA Soil Opinion provides options for the specific protection goals that could be used within a risk assessment to protect the ecosystem services that AMF provide. For most functional services, such as nutrient cycling and soil structure, the manuscript suggests measuring AMF abundance/biomass to be protective. This is in line with the above discussions within this review. It is not suggested that these functional services are measured via an assessment of the provision of a given function. However, it is recommended that, for the protection of biodiversity and genetic and cultural services, AMF need to be protected at the community level. The EFSA Soil Opinion defines this as either the phylogenetic or functional structure of the AMF community. It is paradoxical that functional services are to be protected by measures of biomass but to also consider the inclusion of community functional services provided by AMF to protect biodiversity. Community‐level data are complex and multivariate. Such data are not required within the current PPP ecological risk assessment. The implications of including such data within the risk‐assessment framework are beyond the scope of this review because this is a lengthy discussion worthy of its own article and not limited to AMF but covering all soil microbiota. However, it is important to assess what we currently understand about AMF phylogenetic and functional community structure and why it is unwise to include such metrics within the risk assessment, until such point that we have full comprehension of what these endpoints represent.

Compared to other fungi, AMF are not species‐rich (Lee et al., 2013). However, we have a very poor understanding of how this phylogenetic diversity relates to the functional diversity of AMF. If, because of PPP exposure, the diversity of the AMF community changes within an ecotoxicological test, we need to understand the context of these findings. For example, in the case of an earthworm reproduction test or the measurement of their abundance in a field test, the implications are clear. If their abundance declines, we lose some of the ecosystem services they provide; thus, any PPP causing such effects may fail a risk assessment. Yet, with our current understanding of mycorrhizal ecology, we could not say with any certainty that a difference in the phylogenetic diversity of an AMF community would cause any shift in the function of that community or the services it provides (Van Der Heijden et al., 2004). For instance, Munkvold et al. (2004) found large intraspecific diversity in mycelial growth and phosphorus uptake within *Glomus* spp., illustrating that a low phylogenetic diversity of AMF can maintain large functional diversity. This is supported by Mensah et al. (2015), who show that isolates with the greatest ability to promote plant nutrition were spread across genera and were not specific to any individual species or phylogenetically correlated. Intrafamily variability may still exist for the functional effects on AMF on plant biomass, but Hart and Reader (2002) found no phylogenetic preservation of AMF effects on foliar phosphorus content. Koch et al. (2017) report significant phylogenetic structuring of fungal morphology and growth traits across 56 isolates, 17 genera, and six families; however, this was not linked to AMF functioning, as measured by plant growth responses to AMF, suggesting that this phylogenetic diversity did not lead to functional divergence. Furthermore, in measuring plant growth responses to AMF, Klironomos (2003) shows that the function of AMF may be context‐dependent and that the functional benefit of any given AMF species shifts from parasitism to mutualism, depending on the plant host. Overall, it is evident that there is an uncoupling between the phylogenetic and functional diversity of AMF. Without further understanding of these linkages, we cannot characterize the potential implications of PPPs on the service provision of AMF based solely on phylogenetic data.

In addition, we note that phylogenetic diversity is not defined within the EFSA Soil Opinion. This could be either the general AMF diversity in the soil, which represents the potential pool of AMF with which interactions could be formed, or the intraradical diversity, which represents the AMF actively interacting with the plant host. Currently, it is not known which community would represent the best choice for a risk assessment in terms of sensitivity and ability to produce reliable and informative data for use in the decision‐making process. Further research should seek to understand which compartment of the AMF community is best placed for use within a PPP risk assessment.

Perhaps expectedly, most studies focus on the functioning of AMF regarding their benefit for plant health and nutrition. However, in agroecosystems, the within‐field benefit of AMF to crop yields is not always apparent (Ryan & Graham, 2018; Thirkell et al., 2017). Therefore, the focus may be placed on other aspects of AMF functioning such as their contribution to soil structure and other factors that indirectly benefit plants and crops. However, such services may be incredibly challenging to measure in the context of a risk assessment, but there is potential shown in current research. For example, glomalin and glomalin‐related soil proteins are the proteins produced by AMF that promote the beneficial impact of mycorrhizae on soil structure and aggregation (Holátko et al., 2021; Rillig et al., 2003). Existing methods to quantify soil glomalin content have issues binding to non‐target proteins and soil contaminants, raising concerns about their accuracy (Gillespie et al., 2011; Holátko et al., 2021; Rosier et al., 2006). However, a recent pilot study described the potential to assess the abundance of glomalin genes within soils (Magurno et al., 2019). Although that study was conducted on a single sample, the potential for such approaches to understand AMF community functioning without the need for phylogenetic data is clear. The assessment of AMF functional genes could represent a useful way of incorporating functional data into a PPP risk assessment; however, it is currently untested and requires significant further research investment to uncover its potential, which undoubtedly will be a gradual process.

Overall, we cannot currently understand the significance of phylogenetic shifts in AMF community composition because we do not understand the functional diversity of AMF. Because the risk assessment process is based on a hazard and exposure model, we cannot define the risk if we cannot quantity the hazard. Therefore, it is premature to include AMF phylogenetic or functional data into the risk‐assessment process regarding AMF. There is significant potential in this area to explore future options to develop an AMF risk assessment; however, it is too early in the research and methodological development to advocate for their use. This is not to say that the inclusion of all AMF tests in a risk assessment needs to be dependent on the development of an understanding of AMF phylogenetic and functional diversity. The present review has established that, although there are many unknowns to be addressed, current methodologies could be utilized to incorporate AMF into a fully tiered risk‐assessment process. However, we must be cautious that the reach of the legislation in PPP registration does not extend beyond current ecological understanding.

## CONCLUDING REMARKS

The EFSA Soil Opinion outlines the possibility to include AMF within the ecotoxicological testing of PPPs within a risk assessment. Considering this, the goal of the present review was to examine how AMF can be integrated into a fully tiered risk‐assessment framework, through an exploration of the relevant literature. We have discussed various available in vitro methods that can be utilized in the first tier of a risk assessment, and we conclude that spore germination tests may represent a sensible option if key research questions can be answered. These questions include the need to understand how to integrate fungistatic outcomes into these tests either as an extension to current methods or as a refinement approach, the potential for genetic divergence between different lineages of culture isolates to cause differential outcomes in ecotoxicological studies, and the need to calibrate spore germination test outcomes to real‐world AMF performance metrics to ensure that these tests are protective of the ecosystem services provided by AMF.

The EFSA Soil Opinion does not include detail as to any potential higher‐tier experimental approaches. We have considered how existing methods could satisfy these needs at intermediate and higher tiers. Our discussions have addressed potential refinements of Tier 1 approaches, the use of microcosm and mesocosm studies, and the need for further investigation into how to best include host plants within tests examining herbicidal PPPs. We have suggested potential endpoints for use, including AMF biomass via NLFA testing, and suggested further research into this area, including understanding the variability and usability of this metric between different soils, regions, and agronomic systems. We have also considered the highest tier of a potential future risk assessment: field studies. Key knowledge needs for field studies include how to accommodate the natural variability of AMF communities into validity criteria, the importance of soil selection in providing agronomic relevance, and the need to identify an appropriate toxic reference compound.

Finally, we have considered the suggestion in the EFSA Soil Opinion to incorporate AMF community metrics into the risk‐assessment process. While we acknowledge the desire to include the latest technologies into the PPP ecotoxicology, we caution against the integration of AMF community‐level metrics into the risk assessment. Instead, we need to further our collective understanding of the linkages between the phylogenetic and functional diversity of AMF. Together, there are many unknowns that make it difficult to conclude whether the inclusion of AMF in an ecologically relevant risk assessment is feasible. Only once these uncertainties are addressed can we assess whether the addition of AMF to the PPP risk assessment is ecologically relevant and sufficiently standardizable for regulatory purposes. Finally, as the specific protection goals for AMF have not been finalized, we are currently unable to conclude whether the inclusion of these tests would add to the protection afforded to soil microorganisms and ecosystem services by the PPP risk assessment.

We strongly encourage regulators and researchers, both academic and industrial, to consider the current unknowns surrounding any future introductions of AMF into risk assessment and to establish experiments that will address these knowledge gaps. This requires cross‐party stakeholder engagement. Partnerships between academic and industrial stakeholders can enable this cutting‐edge research to promote the inclusion of relevant, reliable, and robust scientific tests within future PPP policy. Fortunately, such partnerships are being established; for example, the European Union Horizon 2020 ARISTO project (https://cordis.europa.eu/project/id/956496) aims to fill this need, promoting industrial–academic partnerships to assess the ecotoxicological implications of exposure of the soil microbiome, inclusive of AMF, to PPPs. Ultimately, PPPs are a key instrument in the current toolbox of farmers to provide for the increasing demands of a growing population for food, and any risk‐assessment process needs to enable the introduction of newer, safer, more sustainable, and efficacious compounds to the market and ensure access to the safe chemistries required for food security.

## Disclaimer

All authors are employed by companies that research and manufacture plant protection products.

## Author Contributions Statement

**Christopher J. Sweeney**: Conceptualization; Writing—original draft; Writing—review & editing. **Melanie Bottoms, Sian Ellis, Gregor Ernst, Stefan Kimmel, Stefania Loutseti, Agnes Schimera, Leticia Scopel Camargo Carniel, Amanda Sharples, Frank Staab, Michael T. Marx**: Conceptualization; Writing—review & editing.

## Data Availability

No new data were collected for this review.
